# Investigation of serum concentration and the type of opioid used in patients with opium poisoning

**DOI:** 10.1016/j.toxrep.2026.102213

**Published:** 2026-01-31

**Authors:** Armin Mansouri Sarabbadieh, Nazir Fattahi, Mohammad Mehdi Veisi, Afshin Almasi, Zahra Shaahmadi, Toraj Ahmadi Jouybari

**Affiliations:** a Clinical Research Development Center, Imam Khomeini and Mohammad Kermanshahi and Farabi Hospitals, Kermanshah University of Medical Sciences, Kermanshah, Iran; b Research Center for Environmental Determinants of Health (RCEDH), Health Institute, Kermanshah University of Medical Sciences, Kermanshah, Iran

**Keywords:** Opioid poisoning, Methadone, Kermanshah

## Abstract

Opioid poisoning constitutes a major public health issue in Iran and worldwide, with increasing rates attributed to both traditional opiates and synthetic derivatives such as methadone. Reliable assessment of serum opioid concentrations is essential for clinical management and prognosis. Quantitative measurement of serum opioid concentrations determines local epidemiological patterns and identifies risk factors that guide future prevention and treatment strategies. This descriptive cross-sectional study was conducted at Imam Khomeini Hospital in Kermanshah, Iran, from March to June 2023. Fifty adult patients with confirmed opioid poisoning were clinically diagnosed based on the classic opioid toxidrome (decreased consciousness, miotic pupils, and respiratory depression) documented at emergency department admission. Clinical diagnosis was confirmed by positive response to naloxone administration. Demographic and clinical data, type and route of opioid use, clinical features, laboratory results, and patient outcomes were recorded. Serum concentrations of opioids were measured using gas chromatography–mass spectrometry (GC–MS) after administration of antidotes. Statistical analysis was performed with SPSS 26, using a significance threshold of p < 0.05. Among 50 patients (86 % male, mean age 33.5 ± 9.8 years), methadone was the most common cause of poisoning (72 %), followed by opium and heroin. The principal clinical findings included apnea (44 %), miosis (44 %), and weakness/lethargy (38 %), with severe symptoms observed primarily in methadone cases. Serum methadone concentrations were significantly higher in men than women (p = 0.007), and in rural residents compared to urban (p = 0.005). Higher opioid serum levels were associated with more severe clinical presentations and increased need for intensive care. Most patients recovered with medical management; no in-hospital deaths occurred. Methadone has become the leading cause of opioid poisoning in this region, with high serum concentrations predicting increased clinical severity. Measurement of serum opioid levels is a valuable tool for risk stratification and should be integrated into standard management protocols. Attention must be given to both preventive education and tighter regulation of methadone distribution, particularly among high-risk groups.

## Graphical Abstract

**Figure:**
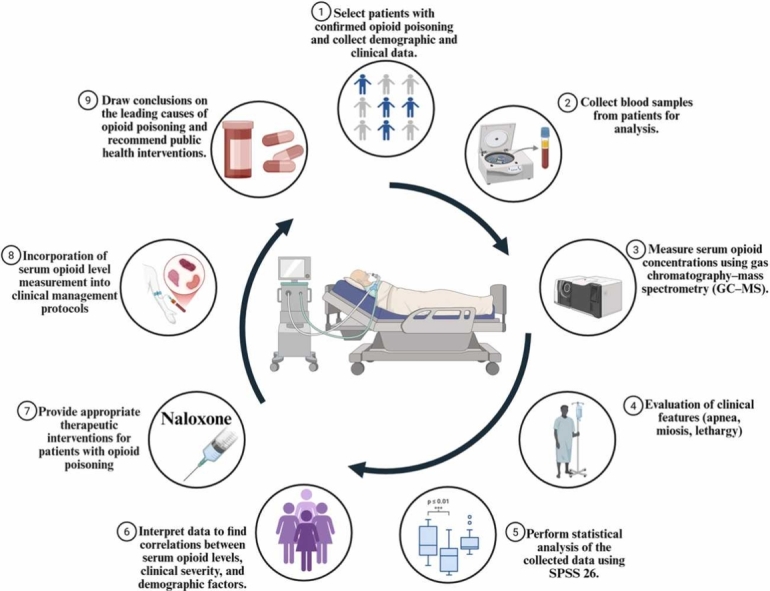

## 1 Introduction

Opioid poisoning has been recognized in recent decades as an increasing public health crisis worldwide, particularly in the Middle East and Iran [1]. This drug group, which includes natural derivatives such as opium and morphine, semi-synthetic drugs like heroin, and synthetic compounds such as methadone and fentanyl, possesses analgesic and sedative properties and is widely used for medical and non-medical purposes [2]. In 2019, approximately 62 million people worldwide used opioids, with about 36.3 million experiencing opioid use disorders [3]. Furthermore, drug use is responsible for approximately 452,000 deaths annually [4]. Iran, due to its geographical location, faces a severe drug use crisis. Having a common border with Afghanistan, the largest opium producer in the world, Iran serves as the primary transit route for drug trafficking to Europe [5]. Drug addiction annually imposes significant economic and cultural burdens on society, and statistics rank it is as the second leading cause of death in the country after road traffic accidents [6]. Approximately 2 million people who use drugs live in Iran, of whom 9–16 % are people who inject drugs [7], [8]. The World Health Organization (WHO) has reported that opium use in Iran is three times higher than the global average [9]. In a study in Tehran, the capital of Iran, drugs were the second most common cause of death (24.8 %), and opium was the most commonly used substance [1].

However, intentional or unintentional opioid misuse can cause acute and life-threatening poisoning [10]. According to global epidemiological studies, the mortality rate due to opioid poisoning in Iran has risen sharply, posing a serious concern for healthcare systems [11]. In recent years, the shift in consumption patterns from traditional substances such as opium to compounds like methadone and tramadol—with the misconception of their greater safety—has been a key factor in rising poisoning cases [12], [13]. Methadone, due to its long half-life and potent central nervous system depression, accounts for the highest rates of severe complications and fatalities [14].

Rapid clinical diagnosis and determination of poisoning severity based on clinical manifestations, laboratory parameters, and physician decision-making are critically important. However, precise serum opioid concentration measurements play a key role in selecting treatments and identifying at-risk patients [15], [16], [17]. Alongside the epidemiological crisis, one of the clinical challenges is the assessment of poisoning severity and patient prognosis [18]. Measurement of serum opioid concentrations in poisoned patients can provide valuable information about the severity of poisoning, type of substance used (methadone, morphine, heroin, or tramadol), and guidance for therapeutic strategies. Yet, in practice, access to such tests, interpretation of values, and determination of risk thresholds remain matters of debate, with limited evidence particularly in populations of developing and underdeveloped countries [19]. Although several studies regarding the epidemiology and clinical complications of opioid poisoning have been conducted in most cities in Iran, most rely on clinical evidence and patient history, infrequently addressing laboratory aspects such as serum drug concentrations [11], [12], [13], [14], [15], [20], [21], [22].

In Iran, especially in western provinces such as Kermanshah, the occurrence of poisoning cases with opium and its derivatives has created many challenges for emergency and treatment centers. However, most studies in this province have been epidemiological and descriptive [23], [24], and there is still insufficient research on serum opioid levels, usage patterns, and complication profiles in Kermanshah province's native populations. Moreover, data regarding differences in serum drug concentrations based on substance type, sex, age, route of administration, and clinical outcomes are lacking. Such data could inform targeted treatment protocols, improve emergency care, and prevent avoidable deaths. This study evaluates serum opioid levels and substance types in poisoned patients—emphasizing correlations among laboratory findings, clinical manifestations, and outcomes—to fill this gap and support targeted regional and national strategies.

## 2 Materials and methods

### 2.1 Study design

The present study is a descriptive cross-sectional study conducted at Imam Khomeini Hospital, Kermanshah, Iran, affiliated with Kermanshah University of Medical Sciences. This study was approved by the Ethics Committee of Kermanshah University of Medical Sciences (No.: IR.KUMS.MED.REC.1402.339) and was conducted in accordance with the principles of the Declaration of Helsinki. Informed written consent was obtained from all participants. The study period covered March to June 2023 (first four months).

### 2.2 Study setting and pre-hospital context

The study was conducted at Imam Khomeini Hospital, a tertiary care teaching hospital in Kermanshah, western Iran. In Iran, emergency medical services (EMS-115) provide ambulance services with paramedics, primarily in urban areas. Pre-hospital naloxone administration is limited and typically available only in major urban centers. Consequently, most patients reach the emergency department via ambulance or family transport without pre-hospital antidote administration.

### 2.3 Data collection

In this study, we included all adult patients (aged 18 years or older) who are admitted during the study period with a definitive diagnosis of opioid poisoning (including, but not limited to, methadone, opium, heroin, and tramadol). The diagnosis of opioid poisoning is made based on clinical assessment and confirmed by laboratory determination of serum opioid levels. Patients were excluded if they had incomplete medical records, were under 18 years of age, presented with non-opioid primary toxicological exposure, or were unable to provide informed consent. Additionally, patients with polysubstance exposure where opioids were secondary, or those for whom no legal representative was available to provide consent, were excluded from the study. Ultimately, using systematic sampling, 50 eligible patients were selected. Data are collected using a standardized case report form. Extracted demographic variables include age, sex, educational level, and place of residence (urban or rural). Clinical data include the type and route of opioid exposure, apparent signs and symptoms (e.g., apnea, miosis, bradycardia), complications, length of hospital stay, treatment methods, and outcome status (complete recovery or discharge with physician approval).

### 2.4 Serum sample collection, preparation and analysis

Serum samples were collected from individuals with opioid poisoning who presented to Imam Khomeini Hospital, Kermanshah. According to the method of Han et al. [25], to remove protein and other substances, 1 mL of serum sample was placed in a test tube, and 0.5 mL of 15 % zinc sulfate solution and 0.5 mL of acetonitrile were added. The test tube was vortexed for 20 min, then incubated for 15 min at 4°C. Finally, the test tube was centrifuged for 5 min at 4000 rpm. The supernatant was collected in another clean tube and diluted with distilled water (to reduce matrix effect) to a final volume of 5 mL, and subjected to the DLLME-DES process. For DLLME-DES, the method of Fatahi et al. [26] was used. Five milliliters of the prepared serum from the previous step was placed in a 10 mL glass test tube, and the ionic strength and pH of the medium were appropriately adjusted. Eighty microliters of DES (extraction solvent) was rapidly injected into the aqueous solution with a gastight syringe (Hamilton, Reno, NV, USA), and vortexed for 20 min. A cloudy solution, resulting from the dispersion of fine DES droplets in the solution, was formed in the test tube. Due to the large contact surface, the analytes were extracted into the DES. Centrifugation separated the organic phase from the aqueous phase and, due to its lower density, collected at the surface of the solution. The test tube was then placed in an ice bath for 5 min to solidify the floating organic droplets. The solidified droplets were then transferred to a conical vial where they rapidly melted at room temperature. Subsequently, 1 μL of the extracted organic phase was injected into the GC-MS using a syringe.

### 2.5 Statistical analysis

Statistical analyses were performed using IBM SPSS Statistics version 26. Continuous variables were summarized as mean ± standard deviation or median (interquartile range) as appropriate. Categorical variables were presented as numbers and percentages. Comparisons between groups were performed using the chi-square test for categorical variables and independent *t*-test or one-way analysis of variance for continuous variables, as indicated. Statistical significance was defined as a two-tailed p-value < 0.05. The analytical approach was chosen to reflect recent practices in opioid poisoning research to ensure comparability and scientific rigor.

### 2.6 Sample size calculation

The sample size for this descriptive cross-sectional study was determined based on the primary outcome of methadone poisoning prevalence among opioid poisoning cases. Sample size calculation was performed using the single population proportion formula:

$$n=\frac{Z^{2}\times p\times (1-p)}{d^{2}}$$

Where Z = 1.96 (corresponding to 95 % confidence level), p = 0.65 (expected proportion of methadone poisoning based on prior regional studies: Hadeiy et al. [27]. reported 58.5 % and Ramezanzadeh et al. [12]. reported 68 %), and d = 0.1 (margin of error of 10 %). This calculation indicated a required sample size of approximately 88 patients. However, due to logistical and temporal constraints—specifically, recruitment limited to a single tertiary center over a 3-month period (March to June 2023)—and considering methodologically similar recently published Iranian studies (Ramezanzadeh et al. [12]., 2024; Saravani et al. [28]., 2024), a pragmatic sample size of 50 patients was deemed acceptable for this preliminary investigation. This sample size was adequate to characterize the epidemiological patterns and serum concentration distributions of opioid poisoning in our setting while providing preliminary data for regional toxicology surveillance.

## 3 Results

Table 1 summarizes the demographic characteristics and types of substances used among the study participants. A total of 50 patients diagnosed with opioid poisoning at Imam Khomeini Hospital were analyzed, comprising 43 men (86 %) and 7 women (14 %), with a mean age of 33.5 ± 9.8 years. The majority were urban residents (90 %), and most had educational attainment below diploma level. Methadone was the dominant opioid identified in 72 % of cases, significantly more prevalent in men than women (p = 0.022). The most common routes of consumption were oral (tablets and syrup) accounting for 92 % combined, with less frequent inhalation.

**Table 1:** Demographic characteristics and type of substance used in patients with opioid poisoning.

**Variable**	**Total (n = 50)**	**Male (n = 43)**	**Female (n = 7)**	p-value*
Age (years), mean ± SD	33.5 ± 9.8	34.0 ± 10.0	30.3 ± 8.3	0.137
Education level (%)				0.769
Below diploma Diploma Associate degree Bachelor’s degree	60 24 10 6	83.3 91.7 80 100	16.7 8.3 20 0	
Residence (%)				0.342
Urban Rural	90 10	84.4 100	15.6 0	
Type of substance (%)				0.022
Methadone Opium/extract blend Tramadol Methadone + Methamphetamine	72 28 4 2	91.7 — — —	8.3 — — —	
Route of consumption (%)				0.771
Oral tablet Oral syrup Inhalation	66 26 8	87.9 84.6 75	12.1 15.4 25	

* Indicates p < 0.05 (statistically significant)

The clinical manifestations, complications, and outcomes stratified by substance type are listed in Table 2. Clinical manifestations predominantly included apnea (44 %), miotic pupils (44 %), weakness/lethargy (38 %), and bradycardia (26 %). Severe symptoms such as apnea and seizures (40 %) were principally observed in methadone-exposed patients, reflecting the higher toxicity and serum levels of this drug. The mean serum concentration of methadone was 74.6 ± 37.7 mg/L. Outcomes were favorable in the majority of cases, with 78 % recovering following medical treatment and 22 % discharged against medical advice. No mortality was reported during the study period.

**Table 2:** Clinical signs, complications, and outcomes according to substance type in patients with opioid poisoning.

**Substance type**	**n**	**Mean serum concentration (mg/L)**	**Apnea (%)**	**Miosis (%)**	**Drowsiness (%)**	**Weakness (%)**	**Bradycardia (%)**	**Seizure (%)**	**No Complications (%)**	**Outcome** **(Recovery / DAMA, %)**
Methadone	36	74.6 ± 37.7	86.4	86.4	45.4	57.9	84.6	40	79.1	78/22
Morphine /opium	10	31.1 ± 39.6	—	—	—	—	—	—	—	—
Tramadol	3	118.8 ± 30.0	—	—	—	—	—	—	—	—
Heroin	1	12.8 ± 10.6	—	—	—	—	—	—	—	—
Total	50	—	—	—	—	—	—	—	—	—

*DAMA=Discharged Against Medical Advice.

Table 3 shows comparisons of serum concentrations according to key demographic and clinical variables. Comparative analysis revealed significant sex differences in serum concentrations of heroin and methadone; men had significantly higher levels than women (p = 0.015 and p = 0.007, respectively). Rural residency correlated with increased methadone levels compared to urban areas (p = 0.005), possibly due to differences in access or dosing patterns. Patients admitted to ICU demonstrated substantially elevated methadone serum concentrations (317.8 ± 24.7 mg/L) compared to non-ICU patients (p = 0.003), confirming the dose-dependent relationship between opioid concentration and clinical severity. Furthermore, there was a positive correlation between methadone levels and length of hospital stay (p = 0.044).

**Table 3:** Comparison of serum concentrations according to key demographic and clinical variables in patients with opioid poisoning.

**Factor/Group**	**Methadone Conc. (mg/L)**	**Heroin Conc. (mg/L)**	p-value*
Male	80.8 ± 36.4	16.4 ± 9.8	0.007/0.015
Female	37.2 ± 20.5	2.0 ± 0.8	
Urban residence	71.8 ± 38.5	—	0.005
Rural residence	101.1 ± 11.6	—	
Full recovery/discharge	84.9 ± 35.1	—	0.002
Discharged against advice	45.3 ± 29.3	—	
ICU admission	317.8 ± 24.7	—	0.003
Poisoning ward admission	78 ± 34.2	—	
Emergency admission	39.2 ± 24.5	—	

* Indicates p < 0.05 (statistically significant); first value is for methadone; second value is for Heroin.

## 4 Discussion

Opioid poisoning remains a significant healthcare challenge worldwide and in Iran, with changing patterns of substance use, clinical manifestations, and outcomes. The present study, based on 50 patients with opioid poisoning admitted to Imam Khomeini Hospital in Kermanshah, provides important information for clinical management and public health policy. Consistent with recent studies in Iran [29], [30], [31] and internationally [32], [33], [34], most patients with opioid poisoning presenting to this center were young males from urban areas. A study in the United States examined the differences between men and women in mortality from opioid and stimulant overdose during the years 2020–2021, and showed that men died at rates approximately 2- to 3-fold higher than women in various opioid categories [35]. According to recent epidemiological forecasting models by Böttcher et al., opioid-related overdose mortality demonstrates a marked age-specific distribution, with overdoses peaking within the 35–40 year age group during the 2015–2019 period, with an emerging trend toward even younger age groups in recent years [36]. Male sex has been identified as a risk factor for opioid poisoning [22]. Research findings published from most provinces of Iran [12], [21], [22], [27], [37] show that over 80 % of poisoning cases occurred in men, with cited reasons including specific characteristics, predisposing histories, and socio-cultural issues that lead to substance abuse. It can be said that men face greater social pressures and have greater access to drugs compared to women, making drug use more likely among them [35], [38]. Additionally, a study by Zarghami in Iran (2015) demonstrated that men predominantly access drugs through peer networks and friends, which is particularly prominent among young men [39]. In a study by Butelman et al. [35] in the United States, it was demonstrated that men, compared to women, have a greater tendency to engage in risky behaviors with complications and higher mortality rates, which may contribute to the higher poisoning rates observed in men.

In our study, methadone poisoning alone accounted for 72 % of cases as the most common opioid used in both sexes, and a significant association was found between sex and type of substance used (p = 0.022). Currently, Iran hosts one of the most extensive methadone maintenance treatments (MMT) programs globally. The MMT program has shown significant positive outcomes in Iran [37]. Nevertheless, concerns about illicit methadone use and increasing numbers of fatal and non-fatal poisoning related to it are rising, and the high prevalence of methadone poisoning in our study despite these programs suggests incomplete understanding of dose escalation, non-prescribed acquisition, and patient compliance. Multiple studies have reported an increased risk of methadone overdose. The poisoning situation in other countries is also consistent with our findings. In France, since 2010, methadone has been the main cause of poisoning as well as opioid-related deaths for many years [40]. A 10-year study in Denmark also showed that in 2012 and 2017, the number of drug users who lost their lives due to methadone was twice as high as other opioids [41]. A study in Canada showed that the illegal market was probably the reason for the increase in methadone poisonings [42]. Soltani Nejad et al. [43] demonstrated in a 10-year study that the rate of acute methadone poisoning and related deaths in Tehran had a marked increase from 43 per one million people in 2000–87.5 per one million in 2010. Hadeiy et al.’s study [27] on substance use patterns showed that between 2012 and 2018, methadone poisoning ranked highest among opioids and their derivatives. Jangjoo et al. [44], [45] conducted a comprehensive study from 2019 to 2023 examining poisoning trends and showed methadone poisoning numbers rose from 1694 to 8873 cases in 2023, and a 6-year study from 2014 in Tehran identified opioids as the leading cause of poisoning, with methadone becoming the predominant cause by the end of the study. This confirms the transformative impact of methadone maintenance therapy (MMT) and the ease of access to methadone in both urban and rural areas of Iran—a phenomenon now cited as the main cause of opioid poisoning admissions in the country. However, it is notable that the incidence of methadone poisoning in our study was higher than several concurrent Iranian surveys, which may reflect regional differences in prescription practices, patient education, and local illegal drug markets.

The spectrum of clinical symptoms observed in patients with poisoning in the present study—characterized by respiratory depression (44 %), drowsiness, weakness, and miotic pupils—is indicative of opioid toxicity, specifically methadone [12]. These findings reflect previous studies where methadone was repeatedly associated with more severe respiratory impairment, prolonged sedation, and higher risk of ICU admission compared to other opioids [28], [43]. The statistically significant correlation between higher serum methadone levels and increased symptom severity, such as apnea and decreased consciousness, supports the clinical utility of quantitative drug level monitoring. In the study by Hon et al. [46] and Wolff [47], miosis was identified as one of the important clinical signs of methadone poisoning [47], [46]. However, in the study conducted by Caplehorn the most common initial manifestations of methadone intoxication were reported to be euphoria, slurred speech, and ataxia [48]. Overall, it should be noted that the clinical presentation of methadone poisoning may encompass a wide range of symptoms, and the occurrence of different signs at the time of admission does not necessarily indicate a contradiction between studies. Rather, such variations may reflect different levels of poisoning severity as well as the varying doses of methadone consumed. Men experienced severe poisoning twice as often as women, and clinical and biochemical outcomes were consistently worse in men. This discrepancy may be explained by deviations from prescribed methadone home doses, since women have been shown to be more likely to adhere to prescribed dosages and are also known to use other drugs such as benzodiazepines with methadone, which can affect serum concentrations and methadone side effects [49], [50]. Most patients in this study fully recovered with prompt medical care. However, a significant minority (22 %) left against medical advice.

The absence of mortality in our study center is encouraging and may be due to early and effective intervention, although other Iranian studies [28], [51], commonly report mortality rates of 5–24 % among poisoned patients admitted to ICUs. However, pre-hospital deaths in the community are not included in this analysis. Additionally, 22 % of patients discharged against medical advice may have experienced subsequent complications or delayed mortality not captured in this study. Therefore, a comprehensive regional assessment would require prospective coordination with all EMS units and health facilities to capture pre-hospital and post-discharge mortality. Measuring serum opioid concentrations was an important aid in clinical assessment in our study and allowed risk stratification based on substance, sex, and outcome. Our observation of significantly higher serum methadone levels in men and rural residents indicates fundamental differences in consumption patterns, dosing errors, and perhaps disparities in health-seeking behavior or pre-hospital delays. The finding that patients requiring ICU care or experiencing adverse effects had the highest serum concentrations highlights the role of laboratory monitoring in guiding acute treatment and informing broader public health surveillance.

## 5 Limitations

The majority of patients were enrolled in methadone maintenance treatment programs, and many subsequently exceeded prescribed doses without medical supervision and obtained additional methadone from unregulated sources. Unfortunately, most patients denied or concealed non-prescribed methadone use, and their family members often lacked awareness of such purchases. Since methadone is a controlled substance in Iran, this distinction is clinically important as risk profiles and clinical outcomes may differ between these groups. This information should be prospectively collected in future studies.

We lacked systematic data on prior opioid use and tolerance status. Patients had varied exposure patterns: chronic methadone users, first-time users, suicide attempts, or those switching from other opioids after abstinence. Since tolerance affects poisoning severity, this information should be prospectively collected in future studies.

Many patients denied use of sedative medications and other substances due to legal concerns, cognitive impairment from acute poisoning, or lack of awareness. This prevented comprehensive documentation of concurrent medication use, limiting our ability to control for potential confounders.

## 6 Conclusion

In our study, we investigated the most common causes of opioid poisoning. This study was the first to measure serum concentrations of the opioids consumed in Kermanshah province. Given that methadone constituted the most significant cause of opioid poisoning among our patients, enhanced monitoring at the MMT centers for drug distribution, supervision of drug use, and providing necessary warnings to patients regarding proper dose adjustment and storage of the medication are increasingly required. These measures could significantly reduce the incidence of poisoning, improve patient outcomes, and inform public health strategies in regions with high opioid use.

## CRediT authorship contribution statement

**Nazir Fattahi:** Methodology. **Mansouri sarabbadieh Armin:** Writing – original draft, Data curation. **ahmadi Jouybari Touraj:** Writing – original draft, Project administration, Methodology, Data curation. **Zahra Shaahmadi:** Writing – original draft. **Afshin Almasi:** Formal analysis. **Mohammad Mehdi Veisi:** Writing – review & editing.

## Ethical approval and participant consent

Ethical approval of this study was obtained from the Ethics Committee of Kermanshah University of Medical Sciences IR.KUMS.MED.REC.1402.339. All procedures were performed in accordance with the relevant guidelines and regulations (Declaration of Helsinki). Informed consent was obtained from all study subjects.

## Ethical approval

Ethical approval of this study was obtained from the Ethics Committee of Kermanshah University of Medical Sciences IR.KUMS.MED.REC.1402.339

## Funding

The research was supported financially by the 10.13039/501100005317Kermanshah University of Medical Sciences.

## Declaration of Competing Interest

The authors declare that they have no known competing financial interests or personal relationships that could have appeared to influence the work reported in this paper.

## Data Availability

Data will be made available on request.
